# Length-Dependent Activation of Contractility and Ca-Transient Kinetics in Auxotonically Contracting Isolated Rat Ventricular Cardiomyocytes

**DOI:** 10.3389/fphys.2019.01473

**Published:** 2019-12-11

**Authors:** Oleg Lookin, Yuri Protsenko

**Affiliations:** ^1^Laboratory of Biological Motility, Institute of Immunology and Physiology, Ural Branch of Russian Academy of Sciences, Yekaterinburg, Russia; ^2^Center for Fundamental Biotechnology and Bioengineering, Institute of Natural Sciences and Mathematics, Ural Federal University, Yekaterinburg, Russia

**Keywords:** rat myocardium, isolated cardiomyocyte, auxotonic contraction, Ca-transient, length-dependent activation, Frank-Starling mechanism

## Abstract

Length-dependent activation (LDA) of contraction is an important mechanism of proper myocardial function that is often blunted in diseases accompanied by deficient contractility and impaired calcium homeostasis. We evaluated how the extent of LDA is related to the decreased force in healthy rat myocardium under negative inotropic conditions that affect the calcium cycle. The length-dependent effects on auxotonic twitch and Ca-transient were compared in isolated rat ventricular cardiomyocytes at room temperature (“25C”) and near-physiological temperature (“35C”) in normal Tyrode and at 25°C with thapsigargin-depleted sarcoplasmic reticulum (“25C + Thap”). At the slack length, a similar negative inotropy in “35C” and “25C + Thap” was accompanied by totally different changes in Ca-transient amplitude, time-to-peak, and time-to-decline from peak to 50% amplitude. End-systolic/end-diastolic tension-sarcomere length relationships were obtained for each individual cell, and the ratio of their slopes, the dimensionless Frank-Starling Gain index, was 2.32 ± 0.16, 1.78 ± 0.09, and 1.37 ± 0.06 in “25C,” “35C” and “25C + Thap,” respectively (mean ± S.E.M.). Ca-transient diastolic level and amplitude did not differ between “25C” and “35C” at any SL, but in “35C” it developed and declined significantly faster. In contrast, thapsigargin-induced depletion of SERCA2a significantly attenuated and retarded Ca-transient. The relative amount of Ca^2+^ utilized by troponin C, evaluated by the integral magnitude of a short-lived component of Ca-transient decline (“bump”), increased by ~25% per each 0.05 μm increase in SL in all groups. The kinetics of the Ca-TnC dissociation, evaluated by the bump time-to-peak, was significantly faster in “35C” and slower in “25C + Thap” vs. “25C” (respectively, 63.7 ± 5.3 and 253.6 ± 8.3% of the value in “25C,” mean ± S.E.M.). In conclusion, a similar inotropic effect can be observed in rat ventricular myocardium under totally different kinetics of free cytosolic calcium. The extent of LDA is not determined by actual peak systolic tension but is regulated by the level of peak systolic calcium and the kinetics of Ca-transient decline which, in turn, are governed by Ca-TnC dissociation and Ca^2+^ reuptake by the sarcoplasmic reticulum. Altogether, these findings constitute new evidence about the role of the length-dependent modulation of Ca^2+^ homeostasis in the mechanisms of calcium regulation of contraction and mechano-calcium feedback in the myocardium.

## Introduction

The length-dependent activation (LDA) of myocardial contractility plays an important role in the adaptation of ventricular systolic pressure to the changes in end-diastolic ventricular volume. LDA constitutes the Frank-Starling Mechanism (FSM) and in normal myocardium is regulated by numerous well-tuned mechanisms, including myofilamental Ca^2+^ sensitivity (Dobesh et al., 2002), coordinated actomyosin interaction (Farman et al., 2011; Milani-Nejad et al., 2015; Zhang et al., 2017), giant protein titin (Cazorla et al., 2001; Fukuda and Granzier, 2004; Li et al., 2019), and the phosphorylation of myosin binding protein C and regulatory protein troponin (Korte et al., 2012; Wijnker et al., 2014; Kumar et al., 2015).

The availability of Ca^2+^ ions to interact with regulatory units of thin filaments is a primary condition for the initiation of a contractile response. While the cooperative activation of contractile filaments itself is independent of the sarcomere length, the Ca^2+^ sensitivity of myofilaments increases with longer sarcomere length (de Tombe et al., 2010). Therefore, dynamic changes of free cytosolic calcium in electrically stimulated cardiac cells (Ca-transient) are vital to how calcium ions will communicate with myofilaments. In contrast, the mechanical state of sarcomeres like prestretch will strongly affect these changes. Environmental factors, both physical ones like temperature and biochemical ones like treatment with a drug, may affect the level of diastolic and/or cytosolic Ca^2+^, for example *via* the ATP-dependent Ca^2+^ pump of the sarcoplasmic reticulum (SERCA2a). These changes in Ca^2+^ homeostasis lead to modifications in the length-dependent activation of contraction (ter Keurs, 2012; Smith and Eisner, 2019). While the FSM can be easily assessed from the force-length relationship under different inotropic conditions, it is still questionable how substantial changes in diastolic or peak systolic Ca^2+^ can modulate LDA.

The length-dependent activation (LDA) of contraction was evaluated in auxotonically contracting rat ventricular cardiomyocytes under three environmental conditions: in normal saline at 25°C (the referent condition), in normal saline at 35°C, and in thapsigargin-containing saline at 25°C. An increase in temperature from 25 to 35°C was chosen to accelerate Ca^2+^ uptake by SERCA2a, which is a main contributor to Ca^2+^ extrusion from the interfilament space (Bassani et al., 1994; Mackiewicz and Lewartowski, 2006). Thapsigargin, in contrast, inhibits SERCA2a (Bassani et al., 1994; Treiman et al., 1998; Bassani and Bassani, 2003) and has an opposite effect on the kinetics of free cytosolic Ca^2+^. It is assumed that both conditions have a minor direct effect on the intrinsic properties of contractile machinery compared to Ca^2+^-induced modulation of contraction. In this study, we compared the extent of LDA in cardiac cells with substantially different levels of Ca^2+^ to answer two main questions: (1) which changes in Ca-transient characteristics underlie length-dependent effects on force generation and (2) is force deficiency under negative inotropic conditions accompanied by the loss of LDA.

## Materials and Methods

This study was carried out in accordance with the principles of the Basel Declaration and recommendations of The Animal Care and Use Committee of the Institute of Immunology and Physiology UB RAS (IIP). The experimental protocol was approved by The Animal Care and Use Committee of IIP. The animals were obtained from the Institutional Animal House and maintained under standard conditions.

### Isolation of Ventricular Cardiomyocytes

Two-month-old male and female Wistar rats were used in this study. The rats were anesthetized with 15 mg/kg zolazepam (Zoletil100®; Virbac, Carros, France), injected by heparin (1,000 U/kg), and were euthanized 15 min later. The isolated heart was cannulated through the aorta to the Whole Heart Perfusion System (Radnoti, AD Instruments, Australia) and Langendorff-perfused by modified Krebs-Henseleit Buffer (KHB, in mM: NaCl 118, KCl 4.7, MgSO_4_ 1.2, KH_2_PO_4_ 1.2, NaHCO_3_ 25, HEPES 10, CaCl_2_ 1.25, glucose 11.1) at 37°C and saturated with 95% O_2_ + 5% CO_2_. After equilibration at the rate of 90–180 beats/min, the perfusion was switched to nominally Ca^2+^-free KHB (25 μM CaCl_2_), and the heart was perfused for 25–30 min more. After that, enzymatic digestion of extracellular tissue was performed by adding collagenase (1 mg/ml Collagenase Type 2, Worthington Biochemical Corporation, USA) to the nominally Ca^2+^-free KHB and perfusion by this media for another 20 min. The digested ventricles were fragmented in nominally Ca^2+^-free and collagenase-free KHB. The cell suspension was transferred to Tyrode (in mM: NaCl 140, KCl 4.7, MgSO_4_ 1.2, HEPES 10, glucose 11.1) and slowly saturated by Ca^2+^ up to the final concentration of 1.25 mM. Ca^2+^-tolerant, well-striated, rod-shaped cells were stained using Ca^2+^-sensitive fluorophore rhod-2 (5 μM in final Tyrode) in its esterified AM-form. All chemicals were purchased from Sigma-Aldrich (St Louis, MO, USA) except for collagenase (Collagenase Type 2, Worthington Biochemical Corporation, USA).

### Carbon Fiber Technique

**Figure 1:**
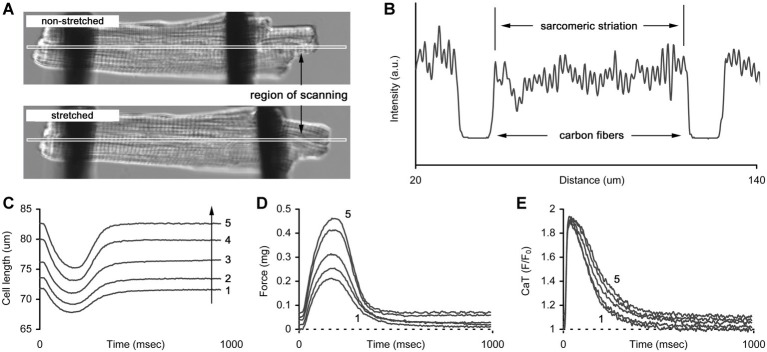
The technique of carbon fibers (CFs) applied to manipulate the length of cardiomyocyte. **(A)** Two images of a cell without and with stretch applied by CFs. The long and narrow region of scanning indicates the area where image acquisition is performed to retrieve cell mechanics and sarcomeric striation. **(B)** The example of intensity profile obtained from a region of scanning. Two areas with lowest intensity correspond to the positions of CFs and the striation pattern *between* the areas is used to retrieve mean sarcomere length. **(C)** The series of auxotonic shortenings obtained at different cell lengths (numbered from 1 to 5, arrow indicates the increase in length). **(D)** The series of auxotonic forces calculated as a product of auxotonic shortening and mean stiffness coefficient of the carbon fibers used in this measurement. **(E)** The series of Ca-transients (CaT, presented as *F*/*F_0_* ratio) measured simultaneously with auxotonic shortenings shown in **(C)**. Numbers in **(C–E)** indicate cell length (1 – slack length, 2…4 – gradual increase in length, 5 – maximal length).

Auxotonic contraction of a cardiomyocyte was measured by a pair of thin carbon fibers (Tsukuba Materials Information Laboratory Ltd., Japan) attached to both ends of a cell and controlled by precise micromanipulators (MP285, Sutter Instrument, USA) and a command unit (ROE200, Sutter Instrument, USA). Contraction and Ca-transient were simultaneously measured by a laser confocal scanning microscope (LSM 710, Carl Zeiss, Germany). Prior to the measurement, a narrow region of scanning was selected on the cell image, assuming it contained both CFs (Figure 1A). Within the region, the intensity profile was reconstructed as a function of distance from the left edge of the image (Figure 1B). The distance between CFs, not the distance between the cell edges, was assumed to be the cell length. Dynamic changes in the CFs’ positions were easily detected by the intensity profile as the areas with minimal intensity and were then converted to cell shortening traces (Figure 1C). Similarly, the mean sarcomere length was derived from the striation pattern of the cell *between* the fibers based on Discrete Fourier Transform, using custom-made software. In this study, only diastolic values of sarcomere length were used further in the analysis of length-related effects on cell mechanics and free cytosolic calcium. The amount of force produced by a cell was calculated as a product of cell shortening and mean stiffness coefficients of the two fibers (Figure 1D) and was converted to tension by dividing by the elliptical cross-sectional area of a cell (*S* = π*d*^2^/12, where *d* is cell width), according to a common assumption that the cell width-to-depth ratio is about 3:1 (Nishimura et al., 2004). Simultaneous with the mechanical measurements, calcium transients (CaT) in the same cell were acquired (Figure 1E).

### Force-Length Protocol

The distance between carbon fibers without any external load being applied was assumed to be the slack end-diastolic cell length (*EDL_0_*). A step-by-step increase in length by several microns (2–5% of *EDL_0_*) was applied until cell detachment occurred from the carbon fibers. Steady-state auxotonic twitches and Ca-transients (obtained as the *F*/*F_0_* ratio; *F_0_* is the fluorescence intensity in the quiescent cell at slack length) were recorded at each new length and ~10 individual twitches were averaged for further analysis. The characteristics of tension/CaT were analyzed as a function of relative end-diastolic cell length (% of *EDL_0_*) or sarcomere length.

Force-length protocols were implemented for separate sets of cells incubated under different environmental conditions: at room temperature (25°C) and near-physiological (35°C) temperatures in normal Tyrode and at 25°C with specific depletion of sarcoplasmic reticulum Ca^2+^-APTase (SERCA2a) by 1 μM thapsigargin in Tyrode. Thapsigargin is a non-competitive inhibitor of SERCA and prevents Ca^2+^ reuptake. For simplicity, these conditions are identified throughout the manuscript as “25C,” “35C,” and “25C + Thap.” The twitches were elicited by electrical stimulation at 1 Hz pacing rate. Each cell was tested only in one condition because it was completely damaged after implementation of the force-length protocol and its removal from the carbon fibers.

### Statistical Analysis

One-way ANOVA was used to evaluate the significance of difference in mean values between environmental conditions (between-group analysis): (1) twitch/Ca-transient characteristics obtained at slack length, and (2) slopes of linear approximation of tension-length relationships and Frank-Starling Gain values. Mann-Whitney *U*-test was used to evaluate the significance of difference between mean values of twitch characteristics measured under three environmental conditions at the same sarcomere length. The differences were considered significant at *p* < 0.05. Data are presented either as mean ± S.D. or mean ± S.E.M. and are stated accordingly. The number of animals involved in the given group is indicated by “*N*,” and the total number of cells in the group is indicated by “*n*.”

## Results

### Auxotonic Twitch and Ca-Transient at Slack Length

**Figure 2:**
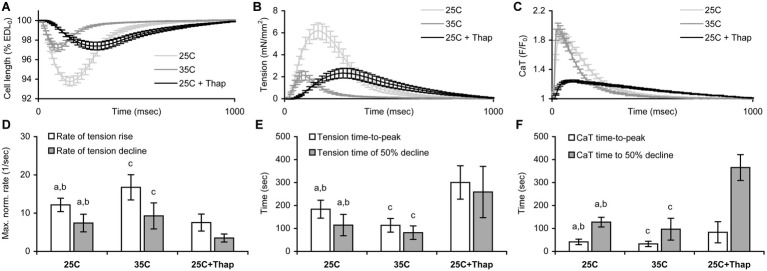
The comparison of shortening, tension, and Ca-transient (CaT) characteristics obtained for isolated rat ventricular cells auxotonically contracting at slack length under different conditions: at room temperature in normal saline (“25C,” *N* = 4 animals, *n* = 34 cells for mechanics, *n* = 32 cells for CaT); at near-physiological temperature in normal saline (“35C,” *N* = 3 animals, *n* = 29 cells for mechanics/CaT); and at room temperature with depleted SERCA2a (“25C + Thap,” *N* = 3 animals, *n* = 27 cells for mechanics/CaT). **(A)** The averaged relative shortening traces. **(B)** The averaged tension traces. **(C)** The averaged CaT traces, presented as the *F*/*F_0_* ratio, where *F_0_* is the fluorescence intensity measured in a quiescent cell at slack length. All traces shown as mean ± S.E.M. **(D)** The maximal rates of tension rise and decline, normalized to active tension. **(E)** The time-to-peak tension and time-to-decline of tension from peak to 50% amplitude (*T_50_*). **(F)** The time-to-peak CaT and time-to-decline of CaT from peak to 50% amplitude (CaT *T_50_*). *EDL_0_* – end-diastolic cell length. Data in **(D–F)** shown as mean ± S.D. ^a^ – significant difference between “25C” and “35C”; ^b^ – significant difference between “25C” and “25C + Thap”; ^c^ – significant difference between “35C” and “25C + Thap” (Mann-Whitney *U*-test, *p* < 0.05).

Figure 2 shows averaged auxotonic shortening and tension as well as the Ca-transient measured in rat ventricular cells at slack length under different conditions. The twitch developed and relaxed faster in cells of “35C” and slower in cells of “25C + Thap” compared to the group “25C” (Figures 2A,B,D,E), for example the maximal rate of tension rise, normalized to active tension, was 16.8 ± 3.3 1/s in “35C” and 7.5 ± 2.2 1/s in “25C + Thap,” compared to 12.2 ± 1.8 1/s in “25C” (mean ± S.D.; all values were significantly different from each other, *p* < 0.05). However, both “35C” and “25C + Thap” cells showed less than one-half of the twitch amplitude in “25C” cells (Figure 2B). Also, a typical effect of an increase in temperature was a substantial acceleration of the Ca-transient in both the rise and decline without changing its amplitude, while SERCA2a depletion led to significant retardation and attenuation of the Ca-transient (Figures 2C,F).

### Length-Dependent Effect on Mechanical Response in Auxotonic Twitch

**Figure 3:**
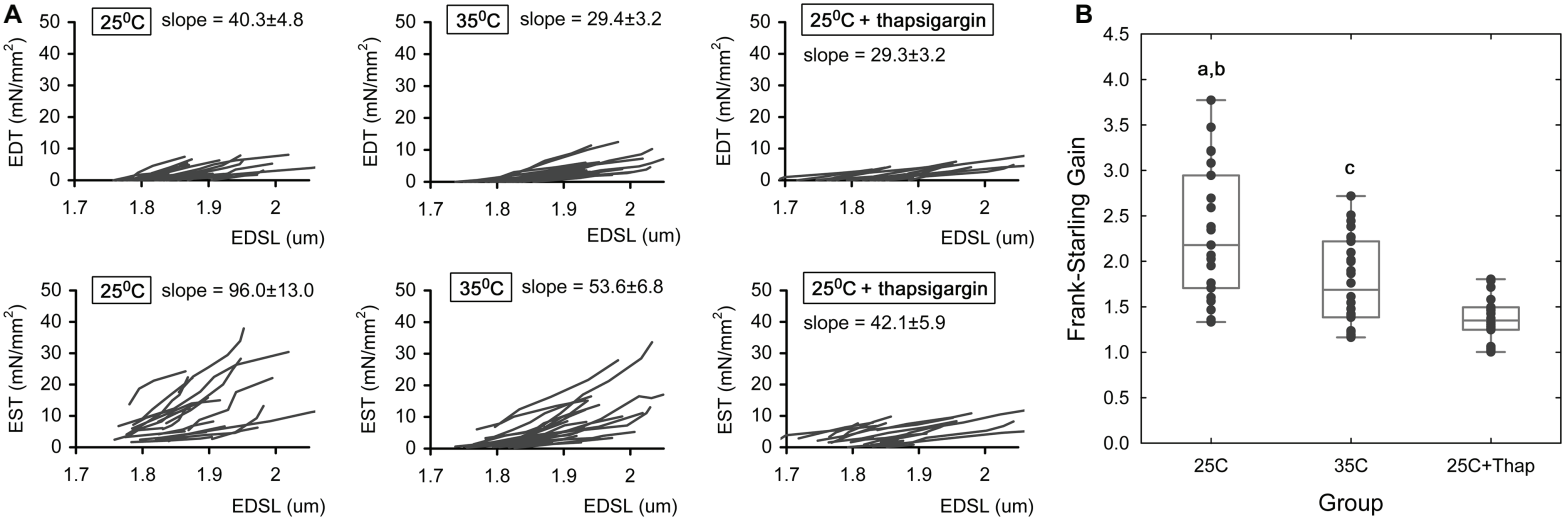
The length-dependent change in active and passive tension in auxotonically contracting isolated rat ventricular cardiomyocytes under different experimental conditions. **(A)** The individual end-diastolic tension – end-diastolic sarcomere length relationships (the top row of panels) and end-systolic tension – end-diastolic sarcomere length relationships (the bottom row of panels) at room temperature in normal saline (“25C,” *N* = 4 animals, *n* = 21 cells); at near-physiological temperature in normal saline (“35C,” *N* = 3 animals, *n* = 26 cells); and at room temperature with depleted SERCA2a by thapsigargin (“25C + Thap,” *N* = 3 animals, *n* = 18 cells). *EDT* – end-diastolic tension, *EST* – end-systolic tension, *EDSL* – end-diastolic sarcomere length. The slopes derived from linear approximation of each individual relationship were averaged and indicated as mean ± S.E.M., units are mN/mm^2^ per one μm of SL. All scales are the same throughout. **(B)** The dimensionless Frank-Starling Gain obtained for the same cells of these groups as a ratio of slopes of EST-EDSL and EDT-EDSL relationships. Scatter-plots of raw data (dots), median value (central horizontal line in the boxes), and 25–75% percentiles are shown. ^a^ – significant difference between “25C” and “35C”; ^b^ – significant difference between “25C” and “25C + Thap”; ^c^ – significant difference between “35C” and “25C + Thap” (Student’s two-sided *t*-test, *p* < 0.05).

To assess length-dependent changes in contractility under different inotropic conditions, auxotonic twitches were measured in cardiomyocytes subjected to a protocol of length change. Sarcomere length (SL) was measured in the diastolic phase. Figure 3A represents the plots of end-diastolic/end-systolic tension vs. end-diastolic sarcomere length (EDT-EDSL, the top row of panels, and EST-EDSL, the bottom row of panels, respectively) obtained in individual cells under different experimental conditions, as indicated in the panels. Linear approximation of each individual EDT-EDSL relationship (Figure 3A, top row of panels) gave mean slope values of 40.3 ± 4.8, 29.4 ± 3.2, and 29.3 ± 3.2 (units are mN/mm^2^ per one μm of SL) for “25C,” “35C,” and “25C + Thap” groups, respectively, in which no significant difference was observed between any of the two groups. SL-dependent potentiation of end-systolic tension was significantly higher in cells of “25C” (Figure 3A, bottom row of panels; the mean slope for the involved EST-EDSL relationships was 96.0 ± 13.0 mN/mm^2^ per one μm of SL) compared to cells of “35C” (mean slope = 53.6 ± 6.8, *p* = 0.004) or “25C + Thap” (mean slope = 42.1 ± 5.9, *p* = 0.001). On the other hand, the mean slopes for “35C” and “25C + Thap” were not significantly different (*p* = 0.234).

As we proposed in our previous study (Bollensdorff et al., 2011), the ratio of slope coefficients of end-systolic/end-diastolic tension-length relationships, or dimensionless Frank-Starling Gain (FSG), is a useful index for evaluating length-dependent activation of contraction. Higher FSG values correspond to a greater length-dependent increase in peak force, i.e., a greater length-induced activation of contraction. This index amounted to 2.32 ± 0.74, 1.78 ± 0.47 and 1.37 ± 0.25 in cells of “25C,” “35C,” and “25C + Thap,” respectively (Figure 3B), in which the mean values were significantly different between any two groups (values given as mean ± S.D., *p* < 0.05). The results show that length-dependent increase in peak systolic tension, according to the Frank-Starling Mechanism, was extremely (roughly 2-fold) attenuated only in cells with depleted SERCA2a (“25C + Thap”), even though the similar extent of negative inotropic effect was observed in “35C” and “25C + Thap” under slack length (see Figure 2B).

As is evident from the data presented in Figure 2, an equivalent depression of systolic tension in “35C” and “25C + Thap” at slack length (compared to the value in “25C”) was not followed by identical changes in timing properties of cell contraction. Similar findings were obtained in the cells subjected to length change protocol, e.g., at any sarcomere length (in the range of 1.8–1.95 μm), the values of time-to-peak tension were substantially smaller in “35C,” but they increased in “25C + Thap,” compared to the values of “25C.” The time-to-decline from peak to 50% of amplitude (*T_50_*) was significantly higher in “25C + Thap” vs. “25C” over the whole range of tested SL, but the significant difference between “25C” and “35C” was found only for SL = 1.8 and 1.85 μm. Nonetheless, neither time-to-peak nor *T_50_* in any group of cells showed dependence on diastolic sarcomere length, so the quantitative difference remained virtually constant during the force-length protocol in which SL was increased from 1.8 to 1.95 μm. On average for this SL range and being compared to the values in “25C” group taken as 100%, the cells of “35C” and “25C + Thap” had, respectively, (1) time-to-peak value of 68.8 ± 4.2 and 157.5 ± 12.6% (values are given here as mean ± S.D.; both are significantly different from “25C,” *p* < 0.05), and (2) *T_50_* value of 90.6 ± 8.0 and 245.0 ± 8.6% (the last value is significantly different from “25C,” *p* < 0.05).

### Length-Dependent Effect on Ca-Transient

**Figure 4:**
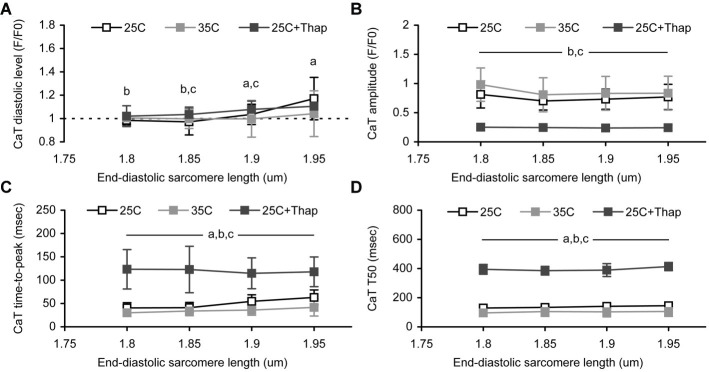
The sarcomere length dependences of Ca-transient (CaT) characteristics obtained in auxotonically contracting isolated rat ventricular cells at room temperature in normal saline (“25C,” *N* = 4 animals, *n* = 21 cells); at near-physiological temperature in normal saline (“35C,” *N* = 3 animals, *n* = 24 cells); and at room temperature with depleted SERCA2a (“25C + Thap,” *N* = 3 animals, *n* = 18 cells). **(A)** The diastolic level. **(B)** The amplitude. **(C)** The time-to-peak. **(D)** The time-to-decline from peak to 50% amplitude (*T_50_*). Data shown as mean ± S.D. Horizontal lines with the significance symbol(s) show the range where this significance is found. ^a^ – significant difference between “25C” and “35C”; ^b^ – significant difference between “25C” and “25C + Thap”; ^c^ – significant difference between “35C” and “25C + Thap” (Mann–Whitney *U*-test, *p* < 0.05).

The deficiency in peak auxotonic shortening/tension observed in “35C” is thought to be related to faster Ca^2+^ removal from cytosol, since the amplitude of Ca-transient (CaT) at any end-diastolic SL was not changed vs. “25C” (Figure 4B), while the diastolic level of Ca-transient was significantly smaller in “35C” vs. “25C” at the sarcomere length of 1.9–1.95 μm (Figure 4A). In contrast, significant depression of active tension in the cells of “25C + Thap” correlates well to the substantially diminished Ca-transient amplitude, which constitutes, on average for SL between 1.8 and 1.95 μm, only 33.0 ± 1.8% of the value of “25C” (mean ± S.D., *p* < 0.05). The diastolic level of Ca-transient was found to gradually increase with SL in “25C” and “25C + Thap” (on average, by ~5.5 and ~2.6% of a value at SL = 1.8 μm per each 0.05 μm increase in sarcomere length), although a minor effect of SL on Ca-transient diastolic level was found in “35C” (Figures 4A,B). This possibly indicates a shallower end-diastolic tension-length relationship under near-physiological temperature vs. those obtained under 25°C (compare the mean slopes in Figure 3A). Note that Ca-transient amplitude did not change with SL in either condition (Figure 4B).

The Ca-transient decline from peak to 50% amplitude was found to be SL-independent in each group of cells (Figure 4D). This possibly reflects the length-independence of Ca^2+^ uptake by SR. However, Ca-transient time-to-peak values were affected by SL in the cells bathed in thapsigargin-free saline (Figure 4C). Both “25C” and “35C” groups showed gradual prolongation of Ca-transient time-to-peak (respectively, by 13.1 ± 11.8 and 10.3 ± 4.2% of the value at SL = 1.8 μm per each 0.05 μm increase in SL, mean ± S.D.), but no such tendency was found in the cells of “25C + Thap” (−1.6 ± 5.0%). This may relate to the different extent of Ca-TnC interaction in cells with normal and reduced peak systolic Ca^2+^ levels, which is discussed below. Interestingly, the mean value of relative peak shortening at slack length in “25C” was >2-fold higher vs. “25C + Thap,” in contrast to Ca-transient amplitude, which was >3-fold higher. Therefore, while the lower extent of shortening-induced inactivation in the cells of the “25C + Thap” group would result in more prolonged Ca-transient at higher SL, as is expected from the length-dependent augmentation of Ca-TnC interaction, the veritable picture is quite the opposite due to the dramatically decreased total Ca^2+^ content in the thapsigargin-bathed cells. Also, absolute values of Ca-transient time-to-peak and *T_50_*, gathered at SL between 1.8 and 1.95 μm, were significantly higher in “25C + Thap” and significantly lower in “35C” compared to the cells of the “25C” group, e.g., because the average for SL in 1.8–1.95 μm, Ca-transient time-to-peak was 71.3 ± 9.5 and 232.1 ± 59.1% of value for “25C” (mean ± S.D.; both are significantly different vs. “25C,” *p* < 0.05).

**Figure 5:**
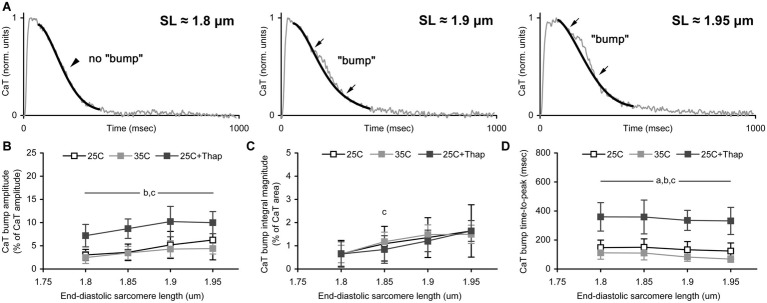
The sarcomere length dependence of Ca-transient (CaT) bump. **(A)** The representative record of CaT traces demonstrating the effect of sarcomere length on the bump extent. Thin gray line shows original CaT trace, thick black line follows the monotonic decline, the bump is the difference between these two lines. Arrows indicate the points of beginning and ending of the bump. Corresponding sarcomere lengths are shown. **(B–D)** The CaT bump characteristics obtained in auxotonically contracting isolated rat ventricular cells at room temperature in normal saline (“25C,” *N* = 4 animals, *n* = 21 cells); at near-physiological temperature in normal saline (“35C,” *N* = 3 animals, *n* = 18 cells); and at room temperature with depleted SERCA2a (“25C + Thap,” *N* = 3 animals, *n* = 18 cells). **(B)** The bump amplitude, as a % of CaT amplitude. **(C)** The bump integral magnitude (the bump area), as a % of area under complete CaT minus this bump area. **(D)** The bump time-to-peak, in msec. Data shown as mean ± S.D. Horizontal lines with the significance symbol(s) show the range where this significance is found. ^a^ – significant difference between “25C” and “35C”; ^b^ – significant difference between “25C” and “25C + Thap”; ^c^ – significant difference between “35C” and “25C + Thap” (Mann-Whitney *U*-test, *p* < 0.05).

To evaluate the length-dependent changes in the kinetics of Ca-TnC interaction in different environmental conditions, an extended analysis of Ca-transient was undertaken. Briefly, a progressive increase in the length of isometrically contracting rat cardiac muscle is accompanied by a concomitant short-lived component over monotonic decline of its Ca-transient [the so-called “bump” (Jiang et al., 1998; Kentish and Wrzosek, 1998)]. Previously, we showed a length-dependence of the bump extent in isometrically contracting rat trabeculae (Lookin and Protsenko, 2019). This analysis of Ca-transient bump characteristics was implemented in this study as well (Figure 5A). The (relative) amplitude of bump was obtained as a % of Ca-transient amplitude, and the (relative) integral magnitude (area) of bump was obtained as a % of the area under Ca-transient trace minus bump area. The time-to-peak of bump was obtained in raw milliseconds calculated from Ca-transient time-to-peak.

The relative amplitude of the bump was significantly higher in cells of “25C + Thap” compared to the “25C” and “35C” groups (Figure 5B) and displayed a gradual SL-dependent change. If the integral level of Ca-TnC interaction was evaluated by the integral magnitude of the bump, there was no significant difference between all groups for any SL (Figure 5C) except SL = 1.85 μm, in which “35C” was significantly higher vs. “25C + Thap” (*p* = 0.025). This indicates that integral Ca-TnC interaction in rat myocardium is highly proportional to sarcoplasmic Ca^2+^ content. Therefore, if contractile machinery of a cardiomyocyte is unchanged (e.g., thapsigargin-inhibited SERCA2a), the relative amount of Ca^2+^ utilized by TnC during the whole twitch is virtually independent of the Ca^2+^ available in cytosol. However, strongly in accordance with the molecular mechanisms of the Frank-Starling Law, the relative bump integral magnitude substantially increased with SL in “25C,” “35C,” and “25C + Thap,” on average by 25.5 ± 12.3, 22.2 ± 22.0, and 26.6 ± 3.8%, respectively, per 0.05 μm increase in SL (values are given as mean ± S.D.; no significant difference was found between any two values). This result shows that acceleration or depletion of SERCA2a has a minor effect on the length-dependent increase of the relative amount of Ca^2+^ utilized by TnC.

Similar to the Ca-transient decline, the rate of bump development was accelerated ~1.5 times in cells of “35C” and decelerated ~2.5 times in cells of “25C + Thap” compared to “25C” (Figure 5D). On average for the tested SL (1.8–1.95 μm), the bump time-to-peak was, respectively, 63.7 ± 9.2 and 253.6 ± 14.4% of the value obtained in “25C” (values are given as mean ± S.D. and significantly differ from the mean value for “25C”). Note that the bump time-to-peak values in actively shortened cells were either SL-independent (as in “25C + Thap”) or showed an SL-dependent *decrease* (as in “35C”), in contrast to the previously observed length-related *increase* in the bump time-to-peak in isometrically contracting muscles (Lookin and Protsenko, 2019).

## Discussion

The length-dependent activation (LDA) of contraction was evaluated in auxotonically contracting rat ventricular cardiomyocytes under three environmental conditions: in normal saline at 25°C (assumed to be the referent state of SERCA2a), in normal saline at 35°C (accelerated Ca^2+^ uptake by SERCA2a), and in thapsigargin-containing saline at 25°C (depleted Ca^2+^ uptake by SERCA2a). The increase in temperature of thapsigargin-free saline affects the rate of Ca^2+^ uptake by sarcoplasmic reticulum (SR) and therefore accelerates Ca^2+^ removal from cytosolic space, thereby providing faster relaxation. Also, it may affect the relative contribution of SERCA, NCX, and PMCA to Ca^2+^ (Mackiewicz and Lewartowski, 2006) and therefore is involved in the regulation of diastolic Ca^2+^ level and contractile function (Shutt et al., 2006). Application of thapsigargin at 25°C had a similar negative inotropic effect but principally different lusitropic effect as well as effects on Ca-transient kinetics, compared to 35°C in normal saline. Thapsigargin, a non-competitive inhibitor of SERCA, prevents Ca^2+^ repletion of SR and markedly attenuates and prolongs Ca-transient (Bassani and Bassani, 2003). These changes in intracellular Ca^2+^ kinetics strongly modulate the contractile response of cardiac cell *via* EC-coupling and mechano-calcium feedback (Eisner et al., 2017). Partial inhibition of SERCA2a by 1 μM thapsigargin was needed to investigate LDA in cells with substantially altered Ca-handling while they were still able to contract.

### Length-Dependent Effect on Mechanical Response and Ca-Transient

The main conclusions of this study are (1) similar negative inotropic effects of certain conditions do not necessarily correspond to the same changes in the level of peak systolic Ca^2+^, (2) the negative inotropic effect is not necessarily accompanied by a deficiency in LDA, and (3) the extent of LDA deficiency is modulated by both the level of peak systolic Ca^2+^ in cytosol and the kinetics of Ca-transient decline. Indeed, an increase in temperature from 25 to 35°C led to a prominent negative inotropic effect with a substantial acceleration of both force and Ca-transient decline. This agrees with previously published findings in rat myocardium (Janssen et al., 2002), but we did not observe accompanying temperature-dependent changes in Ca-transient amplitude. Application of thapsigargin at 25°C led to a negative inotropic effect similar to that found at 35°C in thapsigargin-free saline, but the cells with depleted SERCA2a did show a dramatic decrease in Ca-transient amplitude. Therefore, we conclude that the extent of inotropy is not directly related to the level of peak systolic Ca^2+^, but rather to the dynamic changes in cytosolic Ca^2+^, e.g., the rate of Ca-transient decline. Moreover, although peak tension was attenuated to a highly similar extent in the “35C” and “25C + Thap” groups, LDA was affected by temperature to a modest extent (the Frank-Starling Gain index decreased by <20% of the value at 25°C) compared to the SERCA2a-inhibited cells (the Frank-Starling Gain index decreased 2-fold). Note also that auxotonic contraction is accompanied by shortening-induced inactivation of contractility. It might be expected that the lesser shortening found in the cells of the “35C” and “25C + Thap” groups would intensify their LDA, as measured by Frank-Starling Gain, because these cells contracted under mechanical conditions that are somewhat “closer” to isometric. However, both showed smaller Frank-Starling Gains.

In contrast to length-dependent prolongation of the isometric twitch in healthy rat myocardium (Kentish and Wrzosek, 1998; Janssen et al., 2002; Lookin et al., 2015), the timing characteristics of auxotonic twitch/Ca-transient revealed moderate or minor SL-dependence (for SL = 1.8–1.95 μm) under any experimental condition. This might be closely related to the progressively increased amplitude of auxotonic shortening in a stretched cell and the enhanced shortening-induced attenuation and acceleration of the twitch (Hanft et al., 2008; Iribe et al., 2014). Also, this shortening-induced inactivation of myofilaments may be linked to titin-based modulation of Ca^2+^-troponin interaction (Li et al., 2019) and/or force-dependent recruitment of strong-bound myosin cross-bridges (Campbell et al., 2018). As a result, a positive effect of increased preload and a negative effect of shortening counteract and provide little SL-related variation of timing characteristics in auxotonically beating cells. Indeed, tension and Ca-transient decline times were not affected by SL under all conditions tested in this study, thus supporting the finding that Ca^2+^ removal from cytosol *via* SERCA2a, the dominant player in rat myocardium, is not modulated by SL (Kentish and Wrzosek, 1998). It is interesting that cells with inhibited SERCA2a showed the smallest SL-dependence of Ca-transient timing properties. This may relate to the phenomenon of length-dependent modulation of interaction between Ca^2+^ and troponin C (TnC), e.g., *via* changes in Ca^2+^ sensitivity of the thin filament (Dobesh et al., 2002) or due to the contribution of titin (Fukuda and Granzier, 2004). At normal levels of cytosolic and SR Ca^2+^, Ca-TnC interaction is more intense at larger SL and therefore affects Ca-transient to a greater extent. In cells with reduced Ca^2+^ load to SR (e.g., by thapsigargin), this Ca-dependent modulation of cooperative activation of myofilaments is attenuated, thereby giving negligible SL-dependence of Ca-TnC dissociation. Also, the relatively small effect of SL on Ca-transient decline time supports that SERCA2a can effectively uptake the full amount of Ca^2+^ released from TnC during active shortening. However, such an amount may modulate Ca^2+^ signaling pathways, e.g., nucleoplasmic Ca-transient regulating (Kiess and Kockskämper, 2019) or Ca^2+^-dependent mechanisms essential for LDA in the myocardium (Neves et al., 2016).

The Ca-transient diastolic level was slightly elevated at 25 vs. 35°C at SL = 1.95 μm. Such a discrepancy may be related to the temperature-dependence of SR Ca^2+^ uptake. Ca^2+^ sensitivity of myofilaments is increased with SL and affects the diastolic level of Ca^2+^. Ca^2+^ removal by SERCA2a is accelerated by temperature in rat myocardium (Mackiewicz and Lewartowski, 2006) and prevents a length-dependent shift in diastolic Ca^2+^. The decreased rate of SR Ca^2+^ uptake, e.g. at low temperature or SERCA2a inhibition (Bassani et al., 1994), does not prevent it, similarly to guinea pig myocytes (Le Guennec et al., 1991). This finding contradicts other reports that found length-independence of diastolic Ca^2+^ in rat myocytes at 22–25°C. However, these studies used ~20% lower [Ca^2+^]_o_ (White et al., 1995; Hongo et al., 1996).

Our finding that the relative amplitude and relative integral magnitude of the bump may not follow the changes in the inotropic state in a similar manner (see section “Length-Dependent Effect on Ca-Transient*”* and Figure 5) indicates that careful interpretation of the bump characteristics is needed to speculate about Ca-TnC interaction and its dependence on SL and Ca^2+^ levels. For instance, if a putative Ca-TnC interaction is evaluated during a complete twitch, the integral magnitude of the bump is more reliable vs. the bump amplitude, which reflects a momentary Ca-TnC interaction. A promising finding of this study is that a rate of Ca^2+^ utilization by TnC in auxotonically contracting cells, indirectly obtained from the bump time-to-peak value, shows rather the opposite length-dependence compared to isometrically contracting muscles (Lookin et al., 2015; Lookin and Protsenko, 2019). This is very likely attributed to the shortening-induced inactivation of contraction: increased amplitude of auxotonic shortening in a pre-stretched cell induces more inactivation and faster release of Ca^2+^ from TnC. In contrast, sarcomeres do not shorten during an isometric twitch, and their contractility is regulated mainly by cooperative activation of contractile proteins. Therefore, dynamic changes in length and load continuously regulate Ca^2+^ utilization and redistribution between (intra) cellular components (Hanft et al., 2008; Cazorla and Lacampagne, 2011; ter Keurs, 2012) in which Ca-TnC interaction and Ca^2+^ reuptake by SR are thought to be mostly important in Ca-handling, at least due to their great potency to buffer Ca^2+^ ions (Eisner et al., 2017; Smith and Eisner, 2019). Importantly, the opposite length-related changes in the bump time-to-peak in isometric and auxotonic contractions convincingly support that the bump can be used for indirect evaluation of the kinetics of Ca^2+^ release from TnC during cardiac relaxation.

### The Limitations of the Study

The mechanical response in isolated cardiomyocyte was measured by a carbon fiber technique that was introduced ~30 years ago (Le Guennec et al., 1990) and that is now extensively used (White et al., 1995; Hongo et al., 1996; Iribe et al., 2007; Prosser et al., 2013; Fowler et al., 2015; Helmes et al., 2016; Yamaguchi et al., 2017). The conventional method of two carbon fibers used in this study allowed sarcomere stretch by ~8%. Use of rigid carbon fiber to fix one of the cell ends may provide >10% stretch (Sugiura et al., 2006). Recently, an innovative modification was proposed to pinch each end of a cell using two fibers (Iribe et al., 2014). This improvement gives sarcomere stretch by >15% of the slack length, which is comparable to the effectiveness of biological adhesives like MyoTak™ (IonOptix, USA), which is used to attach a cell to the stretching device (Cazorla et al., 2005; Ait Mou et al., 2008; Prosser et al., 2013). To overcome the problem of the stretch-induced partial detachment of carbon fibers, we used a dimensionless Frank-Starling Gain index to evaluate length-dependent changes in contractility (Bollensdorff et al., 2011).

Another limitation concerns the uncontrollable extent of shortening-induced inactivation in auxotonically contracting cells. Also, since shortening is accompanied by a dynamic change in passive tension, special correction is needed to retrieve the real systolic force (King et al., 2011); similar approach was implemented here. A relatively small extent of shortening-induced inactivation can be achieved during isometric contraction (Iribe et al., 2007). Alternatively, controllable cell shortening, i.e., isotonic/physiological mode, can be used to compare two cells. Therefore, isometric/isotonic/physiological modes are more reasonable for force-length protocols in single cells, although their implementation requires special approaches. At present, only a few studies are available on isolated cells with flow length/load control are available (Iribe et al., 2007, 2014; Helmes et al., 2016) in which fast optical tracking of fibers’ displacement is coordinated with tuned feedback control of their new positions according to the desired mode of contraction.

## Conclusion

The inotropic effects in mammalian myocardium do not correlate simply with the level of peak systolic Ca^2+^ but are governed by dynamic changes in cytosolic Ca^2+^. Totally different kinetics of Ca-transient may be accompanied by negative inotropism to the same extent. Similarly, the extent of length-dependent activation of contraction does not correspond simply to peak systolic tension but is regulated by the level of peak systolic Ca^2+^ and the kinetics of Ca-transient decline. The latter, in turn, is governed by Ca-TnC dissociation and Ca^2+^ reuptake by the sarcoplasmic reticulum. Our data indicate that, at least in healthy rat ventricular myocardium, the integral kinetics of Ca-TnC interaction (association plus dissociation) during a twitch is proportional to the sarcoplasmic Ca^2+^ content. The findings constitute new evidence about the role of length-dependent modulation of Ca-TnC interaction in the mechanisms of calcium regulation of contraction and mechano-calcium feedback in the myocardium.

## Data Availability Statement

The original raw records and datasets generated for this study are available on request to the corresponding author (as well as custom-made software needed to work with raw data).

## Ethics Statement

This study was carried out in accordance with the recommendations of the regulations of the Animal Welfare Act, the National Institutes of Health Guide for the Care and Use of Laboratory Animals, “The Guiding Principles in the Care and Use of Animals” approved by the Council of the American Physiological Society. The experimental protocol was approved by The Animal Care and Use Committee of the Institute of Immunology and Physiology UB RAS.

## Author Contributions

OL and YP contributed to the conception and design of the study. OL performed experiments and data analysis. Both authors prepared the first draft of the manuscript, contributed to manuscript revision, read and approved the submitted version.

### Conflict of Interest

The authors declare that the research was conducted in the absence of any commercial or financial relationships that could be construed as a potential conflict of interest.
